# Chiropractor interaction and treatment equivalence in a pilot randomized controlled trial: an observational analysis of clinical encounter video-recordings

**DOI:** 10.1186/s12998-014-0042-7

**Published:** 2014-12-03

**Authors:** Stacie A Salsbury, James W DeVocht, Maria A Hondras, Michael B Seidman, Clark M Stanford, Christine M Goertz

**Affiliations:** Palmer College of Chiropractic, Palmer Center for Chiropractic Research, 741 Brady Street, Davenport, IA 52803 USA; Institute of Sports Science and Clinical Biomechanics, University of Southern Denmark, Odense, Denmark; The University of Illinois, 801 South Paulina Street, 102c (MC621), Chicago, IL 60612 USA

**Keywords:** Chiropractic, Musculoskeletal manipulations, Observational study, Physician-patient relations, Placebo effect, Randomized controlled trials, Sham treatment, Verbal behavior, Video recording

## Abstract

**Background:**

Chiropractic care is a complex health intervention composed of both treatment effects and non-specific, or placebo, effects. While doctor-patient interactions are a component of the non-specific effects of chiropractic, these effects are not evaluated in most clinical trials. This study aimed to: 1) develop an instrument to assess practitioner-patient interactions; 2) determine the equivalence of a chiropractor’s verbal interactions and treatment delivery for participants allocated to active or sham chiropractic groups; and 3) describe the perceptions of a treatment-masked evaluator and study participants regarding treatment group assignment.

**Methods:**

We conducted an observational analysis of digital video-recordings derived from study visits conducted during a pilot randomized trial of conservative therapies for temporomandibular pain. A theory-based, iterative process developed the 13-item *Chiropractor Interaction and Treatment Equivalence Instrument*. A trained evaluator masked to treatment assignment coded video-recordings of clinical encounters between one chiropractor and multiple visits of 26 participants allocated to active or sham chiropractic treatment groups. Non-parametric statistics were calculated.

**Results:**

The trial ran from January 2010 to October 2011. We analyzed 111 complete video-recordings (54 active, 57 sham). Chiropractor interactions differed between the treatment groups in 7 categories. Active participants received more interactions with clinical information (8 vs. 4) or explanations (3.5 vs. 1) than sham participants within the therapeutic domain. Active participants received more directions (63 vs. 58) and adjusting instrument thrusts (41.5 vs. 23) in the procedural domain and more optimistic (2.5 vs. 0) or neutral (7.5 vs. 5) outcome statements in the treatment effectiveness domain. Active participants recorded longer visit durations (13.5 vs. 10 minutes). The evaluator correctly identified 61% of active care video-recordings as active treatments but categorized only 31% of the sham treatments correctly. Following the first treatment, 82% of active and 11% of sham participants correctly identified their treatment group. At 2-months, 93% of active and 42% of sham participants correctly identified their group assignment.

**Conclusions:**

Our findings show the feasibility of evaluating doctor-patient interactions in chiropractic clinical trials using video-recordings and standardized instrumentation. Clinical trial design and clinician training protocols should improve and assess the equivalence of doctor-patient interactions between treatment groups.

**Trial registration:**

This trial was registered in ClinicalTrials.gov as NCT01021306 on 24 November 2009.

## Background

Chiropractic care is a complex health intervention. Complex health interventions are those healthcare therapies constructed from multiple independent and interacting components rather than composed of a single active ingredient, such as a medication [1–3]. With chiropractic care, these interacting components may include the biomechanical characteristics of spinal or joint manipulation, the therapeutic components of chiropractic care, and the non-specific effects of health interventions in general. The biomechanical characteristics of spinal manipulation [4–8] are commonly described in terms of force-time profile (e.g., loading rates, peak and pre-load forces) [8–10] or the thrust characteristics of location, direction and duration [9,11]. Therapeutic components of chiropractic care may include the underlying theoretical paradigm (i.e., subluxation, biomechanical, or somatic dysfunction) [12–15], specific techniques applied [16–19], and treatment frequency or dose [20]. The non-specific or contextual effects of health interventions are often termed ‘placebo effects’ [3,21,22]. Placebo effects are physiological responses to an intervention that vary by individual and in extent due to the nature of an intervention, its invasiveness, and the patient’s expectations for cure or relief and which may have an impact on patient-reported outcomes, such as pain [23–26]. Placebo effects of a health intervention may include such diverse facets as treatment credibility [3,27], therapeutic ritual [28–30], patient response to clinical observation [28], patient and provider expectations [21,27,31–34], classical conditioning [32,34], the biological pathways involved in pain perception [22,31,32], and patient-practitioner interactions [21,27,31,35].

In clinical trials of chiropractic, manual therapy, acupuncture, medical or surgical interventions, or complementary and alternative medicine (CAM), the notion of the placebo effect may be conflated with the placebo treatment, that is, the comparative or control group [22,23,28,34]. These placebo treatments often are termed ‘sham’ treatments [36,37]. An ideal sham intervention is a procedure that mimics the active treatment in every way except for the absence of the therapeutic component under investigation [23]. Thus, when conducting a randomized controlled trial (RCT) that involves a placebo or sham treatment group, it is not sufficient to provide a sham that is both credible and non-therapeutic [3,21,38,39]. In order to accurately determine the effectiveness of an active treatment, investigators must ensure that non-specific treatment effects (e.g., doctor-patient interactions, time demands, touch or other contact) are the same for participants in the sham group as for the therapeutic group [3,21,23,38,39].

While clinical trials of chiropractic care and other complex health interventions may examine the effects of a treatment on patient-centered outcomes, such as pain or disability [40–42], few trials have considered how placebo effects associated with these therapies may impact patient outcomes [3,21,22,27,43]. One reason researchers have not evaluated placebo effects in clinical trials of chiropractic is the lack of research instruments or data collection processes to quantify these effects. The overall purpose of this observational study was to assess the feasibility of quantifying doctor-patient interactions in sham-controlled chiropractic clinical trials. We also compared these findings to participant perceptions of their treatment group assignment from that same trial. Thus, our specific aims were fourfold. First, we developed a theory-derived data collection tool, the *Chiropractor Interactions and Treatment Equivalence Instrument* (CITE-I), to assess video-recordings of clinical encounters between doctors of chiropractic (DCs) and chiropractic patients. Secondly, we evaluated the equivalence of one chiropractor’s verbal interactions and treatment delivery for participants randomized to the active treatment and sham-controlled chiropractic care groups in an expertise-based, pilot RCT of Activator Methods Chiropractic Technique (AMCT) for temporomandibular disorder (TMD) [44]. Next, we described the video evaluator’s masked assessment of participant treatment assignment with the RCT participants’ beliefs about their treatment group assignment. Finally, we described participants’ perceptions of their treatment group assignment after the first treatment visit and following 2 months of treatment.

## Methods

We conducted an observational analysis of digital video-recordings derived from study visits with participants who received an active or sham chiropractic treatment during a pilot RCT of 4 conservative therapies for TMD-related jaw pain. A theory-based, iterative process developed the 5-domain, 13-variable, *Chiropractor Interaction and Treatment Equivalence Instrument*. In these methods, we describe the design of the pilot RCT, video-recording procedures, the instrument development process, and data collection and analysis procedures.

### Pilot RCT design

The institutional review boards of the Palmer College of Chiropractic, Davenport, Iowa (Approval Number 2009D121), and The University of Iowa, Iowa City, Iowa (Approval Number 200808726) approved the study protocol and human research participant protections for the pilot RCT. This trial was registered in ClinicalTrials.gov as NCT01021306 on 24 November 2009. The trial began January 2010, with data collection completed October 2011. The methods and results of the pilot RCT were described elsewhere [44]. Participants had at least a 6-month history of jaw pain consistent with chronic myofascial TMD. Eighty participants were randomly allocated to one of four treatment groups: active AMCT (n = 20), sham AMCT (n = 19), dental reversible inter-occlusal splint therapy (RIST) (n = 20), or self-care only (n = 21). Participants in all four groups received a basic self-care training module of relaxation, stretching and self-awareness pain modulation therapy. The self-care treatment group received this module, alone. Participants signed a written informed consent. The informed consent document instructed participants that they may be randomized to a “placebo treatment group” with treatments similar in appearance to AMCT and that the investigators did not expect the TMD condition of participants assigned to this group to worsen over the course of the study [23,38]. The consent document informed participants that study visits would be video-recorded to evaluate the doctor’s interactions with participants and that these recordings would not be destroyed.

One DC with over 20 years of experience using the AMCT protocol provided the intervention to all participants in both the active and sham AMCT groups. The DC delivered both treatments with a hand-held, spring-loaded device – the Activator Adjusting Instrument (AAI) (Activator IV, Activator Methods International Ltd., Phoenix, AZ) instead of a manual thrust common to many forms of chiropractic spinal manipulation [17,45]. The DC mimicked the active AMCT protocol for the sham group by using a detuned AAI that made just a sound (like the active AAI), but delivered no thrust. The DC delivered the AMCT protocol, including treatment to the full spine, extremities, and temporomandibular joints for participants in both groups [44]. The DC also performed a gentle occipital stretching procedure following delivery of the standard AMCT treatment. Training on the study protocol emphasized the DC should offer the same type of verbal communication and spend a similar amount of time with patients in each treatment group, including in self-care instruction, examination and testing procedures, and treatment delivery [44].

All participants randomized to the AMCT groups were to receive 12 study visits over 2 months [44]. Primary outcomes included an 11-point numerical rating scale for TMD-related pain [46] and the 14-item Oral Health Impact Profile (OHIP-14) [47] to assess quality of life at 2 months and 6 months. Participant ratings of treatment believability were gathered for all 4 treatment groups following the first and twelfth study visits [19]. Participants also answered the following statement on a 5-item scale (‘strongly believe’ to ‘do not know’): “There are two types of treatments in this research study: active and inactive (placebo). Please indicate which type of treatment you believe you are receiving”. Participant responses for ‘strongly believe’ and ‘somewhat believe’ for active treatment and ‘strongly believe’ and ‘somewhat believe’ for inactive (placebo) treatment were combined in this analysis.

### Video recording and handling process

The study protocol included video recordings of each chiropractic study visit. Thus, our study sample was the video-recorded observations of participant study visits, and not the participants themselves. A digital video-camera (Panasonic model HDC-H520; Newark, NJ, USA) was set up on a tripod in a corner of the treatment room before the participant entered. A card with the participant identification (ID) number and current date was placed in front of the video-camera and recorded for a few seconds. The video-camera was to be positioned to visualize the participant’s entire body lying on the treatment table (from crown of head to feet), as well as the DC as he moved around the table delivering the study treatment. The clinic receptionist used a remote control unit to begin the video-recording process as the participant entered the treatment room and to stop the recording when the participant left the treatment room. The video files were copied from the camera to an external hard drive and named with the participant ID number and recording date. No other identifying information was recorded to maintain participant confidentiality. A study co-leader (JWD) copied the video files from the external hard drive in the chiropractic clinic to a second external hard drive for data transfer to the research center. The HD video-recordings were converted from *.m2ts to *.mp4 files using Roxio Toast Titanium 10 software (Corel Corporation, Ottawa, Ontario, Canada). This version of the video-recordings was stored at the research center on a password-protected computer for long-term back-up and data analysis.

### Instrument development process

Four team members developed the assessment instrument and data collection process to codify the doctor-patient interactions during the chiropractic visits (see Author Information for respective contributions). Team members remained blinded to participants’ treatment assignment throughout the instrument development, data collection and analysis processes. The instrument development process went through 3 primary stages as described below.

#### Stage 1: Preliminary video-recordings review and research question

Two researchers (JWD, SAS) jointly reviewed several video-recordings to examine various aspects of the doctor-patient interactions such as verbal communications (i.e., clinician utterances, participant replies), non-verbal behaviors, and contextual effects (e.g., social interactions, humor, or use of touch) as well as factors related to the recording process to identify the initial coding framework and the strengths and limitations of these video-recordings as data sources. For example, most of the video-recordings did not visualize the participant during pre-treatment consultations or post-treatment interactions due to camera position. In addition, the audio-track often did not record the participants’ side of these conversations clearly. The treatment table muffled participants’ voices when lying prone during much of the AMCT-protocol. Similarly, the camera position for many video-recordings did not allow complete visualization of the non-verbal behaviors (e.g., facial expressions, body position, treatment delivery, AAI positioning) of the DC when his back was to the camera, nor could participants observe these doctor behaviors when they were lying prone. Further, the participants’ ideal body position (i.e., from the crown of the head to the feet) was captured in only about 25% of the videos recorded.

Based on such contextual factors, the investigators (SAS, JWD, MAH) concluded that an analysis of doctor-patient interactions could neither focus on the non-verbal communications of the DC nor emphasize participants’ verbal responses. However, we noted the recordings captured most of the chiropractor’s verbal utterances as well as the “clicking” sounds produced by the thrust of the active and detuned AAIs. A previous study using AAI as a placebo treatment noted this clicking sound supported patients’ assessments of treatment credibility [37]. The team then focused the research question and instrument development process on quantification of the equivalence of the DC’s verbal communications and AAI delivery between the active and sham AMCT groups.

#### Stage 2: Construct identification and instrument development

Literature reviews identified published instruments available for the assessment of doctor-patient interactions in medical encounters [48–50]. Among these, the Roter Interaction Analysis System (RIAS) was identified as the most widely used method of analyzing patient-provider interactions during healthcare encounters [49], and served as the theoretical framework from which our instrument was derived. The RIAS classifies medical communications into two conceptual categories - the socioemotional and task dimensions [49]. While the RIAS has excellent psychometric properties [49], a major limitation of this instrument for an analysis of patient-provider communications within the context of clinical research is that conversational styles of communication in RCTs differ from those in naturally-occurring medical encounters in important ways [30,35,51]. In routine clinical practice, physicians may tailor patient education, advice and support to the individual needs of the patient [27]. In contrast, the communications from the research clinician to the research participant within an RCT is a protocol-driven, or scripted, conversation to minimize its influence on treatment outcomes [28,35,43]. In addition, while research participants are masked to their treatment assignment at the start of a RCT, clinician behavior may lead them to identify whether they are receiving an active or placebo/sham treatment [27,30,35,37,43,51,52]. Thus, the clinician’s verbal communications and treatment delivery should not unmask participants to treatment assignment [43]. Finally, the research clinicians’ verbal interactions may directly impact outcomes assessments in an RCT should the doctor communicate any observed or perceived changes in health status, such as an improvement or decline, to participants [43,51,53].

At this stage, the team first focused instrument development on two theoretical categories of the RIAS: socio-emotional, or ‘care-oriented’ , communications and instrumental, or ‘cure-oriented’ , communications [49,54] to assure these key features of doctor-patient interactions were identified. Video-recordings were viewed over several team meetings to identify how these thematic constructs were expressed by the clinician during treatment. Each member coded the video-recordings using a paper copy of the current assessment form. Video reviewers placed a hash mark in the appropriate cell for each utterance from the clinician and any clicks from the AAI thrust. An utterance was defined as any verbalization that expressed a single idea to a participant. Thus, a sentence in which the DC directed the participant to “turn your head to the right, and to the center, and to the left” would equate 3 unique utterances. Team members stopped the video-recording frequently to discuss how each had categorized the various utterances, the rationale for such categorization, and sought consensus on each classification.

The team reviewed 2–4 video-recordings per session, determined categorical or definitional revisions, and identified form changes. For instance, clinician utterances on participants’ health status (i.e., need for more or fewer adjustments since the last visit) required an added domain for “treatment effectiveness” with optimistic, pessimistic and neutral statements on patient outcomes constituting key variables. This category was of particular importance within a sham-controlled trial where verbal indications of treatment effectiveness may increase participant expectancies for future response [31], serve as a conditioning protocol [31], and impact patient outcome measures [31]. We included a variable for the duration of the study visit to assess whether the clinician spent an equivalent amount time with participants in each group. We also added a tally of AAI clicks (an auditory stimulus that may condition the participant and increase the placebo response [31]) as a rough indicator of the ‘dose’ delivered of active or sham AMCT.

#### Stage 3: Process pre-testing, evaluator training and instrument refinement

SAS and JWD evaluated video-recordings until the team members achieved consensus on the instrument domains, variables, examples and data collection format as no new categories were identified with additional video-recording reviews. MAH confirmed the completeness of initial data collection form. While the team did not assess inter-rater agreement using formal statistics, comparison of categorical totals at the end of each data collection session revealed a high level of agreement between reviewers, with most categories tallying within 1 [for categories with low tallies (0–8 hash marks), such as treatment effectiveness] to 3 points [for categories with high tallies (30–50 hash marks) such as directions or AAI clicks] for each evaluator.

During pre-testing, the team also identified treatment duration differences, with the first treatment visit (T1) lasting 10–20 minutes longer than subsequent study visits (T2-T12). During the T1 visit, the DC spent considerable time discussing the participants’ past medical history, the study protocol, and follow-up activities. As these visits appeared tailored to the individual participant, and differed considerably in duration and content from the other treatment visits, we decided not to include these visits in the analysis for this study.

The team member (MBS) who served as the video evaluator was trained on the video analysis instrument. As in previous coding rounds, team members coded the recordings as a group and discussed unclear utterances, variable definitions and examples. Early in the training, the video evaluator had categorical inconsistencies (primarily with therapeutic domain variables) that were resolved through these discussions. The instrument was reorganized so the most used variables (*clinical information, directions, Activator clicks)* were placed at the top of the grid. Categorical tallies after each coding round noted few differences between the team members.

**Figure 1:**
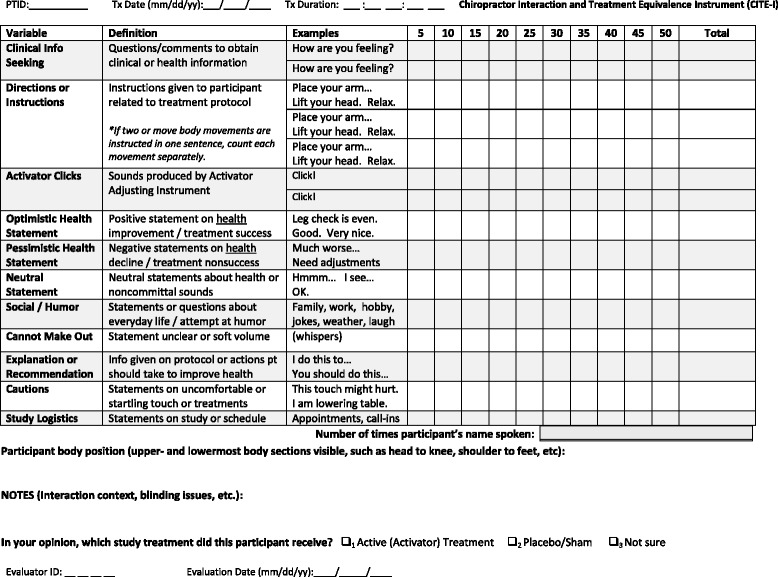
**Chiropractor Interaction and Treatment Equivalence Instrument (CITE-I).**

The team members reviewed and accepted the Chiropractor Interaction and Treatment Equivalence Instrument (CITE-I) for use in the interaction equivalence study. This version of the CITE-I included 5 domains with 13 variables. The affective domain consisted of 2 socio-emotional variables [49,54] categorizing the clinician’s verbal interactions as *social/humor* or *name use*. The therapeutic domain included 3 instrumental variables [49,54]: *clinical information*, *explanations*, and *logistics*. The procedural domain consisted of 3 variables addressing treatment implementation and fidelity [55] including adherence, delivery, and dose: *directions, cautions,* and *Activator clicks,* or the sound produced by the adjusting instrument. The treatment effectiveness domain categorized *optimistic, pessimistic* and *neutral* statements about health or treatment outcomes [31]. Lastly, the encounter context domain tabulated the *duration* of the treatment encounter as an additional measure of dose, as well as any *unclear statements* made by the clinician that the video evaluator could not definitively place into another category. The CITE-I also included a field to denote how much of the participant’s *body position* was on the video and a *notes field* to record additional details of the interaction context, blinding issues, etc. The final item on the CITE-I asks the video evaluator to denote which study treatment he believed the participant to have received (active, placebo/sham or not sure). Figure 1 presents the CITE-I instrument including variable definitions and examples.

### Data collection

One team member (MBS) evaluated the video-recordings of the chiropractic visits using the CITE-I. A flash drive of video-recordings included mixed participants from discontinuous study visits to assure the evaluator did not view an entire treatment series sequentially. The evaluator viewed the recordings while wearing headphones to minimize external distractions. When necessary, portions of the video-recordings were replayed to enhance the accuracy of data collection. This process was repeated until all video recordings were evaluated.

### Data management and data analysis

Completed CITE-I forms were submitted to the Office of Data Management for double key entry into an electronic spreadsheet once all video-recordings in an analytic set were evaluated. Tally marks were counted twice and entered as a total for each category by the evaluator, with these sums double checked by data entry personnel. Data were organized by participant ID number, treatment date, and treatment visit number. Participant treatment believability items were data entered at the time of the pilot RCT. Data were analyzed using the SAS statistical analysis software package (Version 9.2, SAS Institute Inc., Cary, North Carolina, USA). We report simple descriptive statistics (median, interquartile ranges [IQR], and/or number and percentage) to characterize our sample of video-recordings. Formal statistical tests of significance were not appropriate at this stage of instrument development as our primary aim was to assess whether video-recordings were a feasible means of evaluating doctor-patient interactions and not to test hypotheses based on those interactions.

## Results

### Video-recording evaluation flowchart

**Figure 2:**
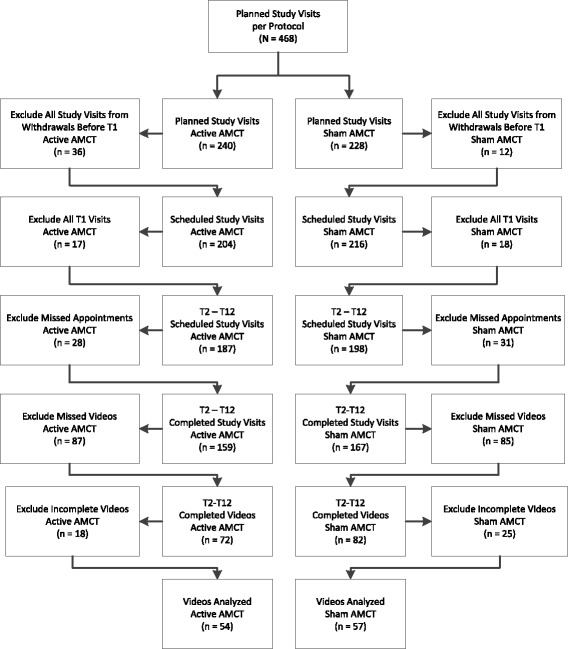
**Video-recording flowchart.**

Figure 2 presents a flowchart of the video-recordings evaluated for this study. Each participant allocated to a chiropractic group (n = 39) was to receive 12 visits to the chiropractor per study protocol (n = 468). An equal number of participants from each group (n = 13) had at least 1 video-recording reviewed for this study. Four participants (3 in active AMCT, 1 in sham AMCT) withdrew from the trial before the first treatment, while 9 participants (5 in active AMCT, 4 in sham AMCT) did not have any video-recordings made during the trial. The mean number of video-recordings completed for all participants was 4.4 (range from 0–11).

For this analysis, we excluded all T1 visits from this analysis due to their longer durations and the more personalized nature of the encounters as compared to the T2-T12 visits. Other video-recordings were either not available or incomplete and not included. Of these, the number of missed appointments (n = 59), missed video-recordings (n = 172), and video-recordings excluded due to incomplete recordings (n = 43), either from video-recordings that began after the visit was in progress or that ended before the visit concluded, were equivalent between groups. In total, we analyzed 24% (111/468) of the planned active and sham AMCT study visits in this pilot RCT. The video evaluator coded 54 video-recordings from 13 active AMCT participants and 57 video-recordings from 13 sham AMCT participants for this analysis.

### Chiropractor interactions and treatment equivalence

Table 1 presents results for the video analysis of clinician interaction and treatment equivalence between active and sham AMCT groups. Five categories, *clinical information*, *explanations*, *directions, optimistic statements*, and *neutral statements* revealed notable differences in the DC’s verbal interactions, while two categories, *Activator clicks* and *encounter duration,* denoted disparities in treatment equivalence between the active and sham AMCT groups.

**Table 1:** **Video-analysis of chiropractor interaction equivalence between active and sham Activator Methods Chiropractic Technique (AMCT) treatment groups**

			**Active AMCT**		**Sham AMCT**	
			**(n =54)**		**(n =57)**	
**Domain**	**Variable**	**Definition**	**Median (SD)**	**IQR**	**Median (SD)**	**IQR**
**Affective**	**Social/Humor**	Statements or questions about everyday life or attempts at humor	9.0 (14.9)	14	8.0 (11.2)	13
	**Name Use**	Number of times the name of the participant was spoken	2.0 (1.3)	2	2.0 (1.5)	1
**Therapeutic**	**Clinical Information**	Questions or comments to obtain clinical or health information	8.0 (5.3)	7	4.0 (3.1)	4
	**Explanations**	Information on protocol or actions to take to improve health	3.5 (9.2)	8	1.0 (3.6)	3
	**Logistics**	Statements on study procedures or treatment schedule	1.0 (1.4)	2	2.0 (2.5)	1
**Procedural**	**Directions**	Instructions given related to treatment protocol	63.0 (11.6)	15	58.0 (6.7)	8
	**Cautions**	Statements on uncomfortable or startling touch or treatments	3.0 (1.5)	2	3.0 (1.2)	1
	**Activator Clicks**	Sounds produced by Activator Adjusting Instrument	41.5 (30.1)	44	23.0 (10.1)	13
**Treatment Effectiveness**	**Optimistic**	Positive statement on health improvement or treatment success	2.5 (4.0)	6	0 (1.6)	0
	**Pessimistic**	Negative statement on health improvement or treatment success	0 (1.1)	1	0 (0.7)	0
	**Neutral**	Neutral statement about health or noncommittal sounds	7.5 (4.1)	6	5.0 (3.6)	4
**Encounter Context**	**Encounter Duration**	Duration of treatment in minutes	13.5 (4.1)	5	10.0 (1.8)	2
	**Unclear Statement**	Statement unclear or spoken at soft volume	2.0 (2.6)	3	1.0 (2.0)	3

Within the therapeutic domain, the participants in active AMCT had twice as many verbal interactions where the clinician sought *clinical information* than did sham AMCT participants (median 8.0 vs. 4.0 per visit). Active AMCT participants also received more *explanations* on the study protocol or recommendations on actions to take to improve health compared to sham AMCT participants (median 3.5 vs. 1.0 per visit). Statements about study *logistics* favored sham ACMT participants over active AMCT participants (median 2.0 vs. 1.0 per visit).

Within the procedural domain, active AMCT participants received more *directions* from the clinician than did sham AMCT participants (median 63 vs. 58 per visit). Active AMCT participants also received more *Activator clicks* than did sham AMCT participants (median 41.5 clicks vs. 23 clicks per visit). *Cautions* were similar between groups.

Within the treatment effectiveness domain, the DC offered active AMCT participants more *optimistic statements* about health improvements or treatment success than participants in the sham AMCT group (median 2.5 comments vs. 0 comments per visit). Active AMCT participants also received more *neutral statements* about their treatments than did sham AMCT participants (median 7.5 comments vs. 5 comments per visit), while few *pessimistic statements* were offered to participants in either treatment group.

Within the encounter context domain, the mean *encounter duration* was somewhat longer for the active AMCT group than the sham AMCT group (13.5 minutes vs. 10.0 minutes per treatment). More *unclear statements* were recorded for the active AMCT group (median 2.0 versus 1.0). Neither of the affective domain variables (*social/humor* or *name use*) differed appreciably between the treatment groups.

### Treatment group assignment evaluation

Table 2 presents the results of the masked assessment of treatment assignment by the video evaluator and compares these data to participant’s perceptions about their treatment assignment. The video evaluator correctly assigned an assessment of ‘active treatment’ to 33 (61%) of the active AMCT video-recordings, with most of the remaining (n = 17; 31%) video-recordings receiving a ‘not sure’ designation. The video evaluator assigned an assessment of ‘active treatment’ (n = 16; 28%), ‘placebo/sham’ (n = 18; 31%), and ‘not sure’ (n = 22; 39%) to the sham AMCT video-recordings.

**Table 2:** **Video evaluator assessment of treatment assignment compared to participant treatment believability ratings**

		**Active AMCT Videos**		**Sham AMCT Videos**	
		**(n =54)**		**(n =57)**	
**Variable**	**Response**	**n^**	**%**	**n^**	**%**
Video Evaluator Assessment of Treatment Assignment	Active AMCT	33	61	16	28
	Sham AMCT	3	6	18	31
	Not Sure	17	31	22	39
		**Active AMCT participants**		**Sham AMCT participants**	
		**(n =17)**		**(n =18)**	
**Variable**	**Response**	**n**	**%**	**n**	**%**
Participant Believability 1st Treatment Visit	Active Treatment^+^	14	82	12	66
	Inactive Treatment (Placebo)^+^	1	6	2	11
	Do Not Know	2	12	4	22
		**Active AMCT participants**		**Sham AMCT participants**	
		**(n =14)**		**(n =14)**	
**Variable**	**Response**	**n**	**%**	**n**	**%**
Participant Believability Month 2	Active Treatment^+^	13	93	8	58
	Inactive Treatment (Placebo)^+^	1	7	6	42
	Do Not Know	0	0	0	0

+Strongly believe and somewhat believe were combined for presentation.

^Missing data.

In contrast to the treatment-masked evaluator, study participants more readily identified their treatment group assignments, particularly those in the active AMCT group. After the first study visit, 82% (n = 14) of active AMCT participants rated their treatment as an ‘active treatment’, with 6% (n = 1) rating the treatment as inactive or placebo, and 12% (n = 2) stating they did not know which treatment they received. After their first treatment, 66% (n = 12) of sham AMCT participants rated their treatment as active, 11% (n = 2) as inactive or placebo, and 22% (n = 4) as did not know. At the 2-month assessment, participant ratings of ‘active treatment’ increased to 93% (n = 13) for active AMCT participants. For sham AMCT participants, active treatment ratings dropped to 58% (n = 8), with inactive or placebo ratings increasing to 42% (n = 6). No participant in either group stated they did not know their treatment group at the 2-month evaluation.

## Discussion

To our knowledge, this study is the first to assess the equivalence of verbal interactions and treatment delivery for a doctor of chiropractic providing active and sham chiropractic interventions within the context of a randomized controlled trial. Many studies of spinal manipulation or other chiropractic therapies have used sham adjustments as a comparator [36,56], including those using a detuned Activator adjusting instrument as the sham [37,57]. Researchers who conduct sham or placebo-controlled trials of complementary therapies, including chiropractic, have espoused the need for the standardization of the non-specific aspects of treatment, including treatment duration and the interventionists’ verbal and non-verbal communications, between study groups [37,52,58]. And yet, most have evaluated only patient perceptions of the believability of the sham or their success in masking treatment assignment [37,52,59–61]. Few studies, if any, of chiropractic interventions have discussed the potential placebo effects derived from the doctor’s interpersonal interactions with patients.

This study showed the feasibility of quantifying the verbal interactions and treatment equivalence of chiropractors within a clinical trial using a standardized data collection process. This finding has relevance for future clinical studies. Our data collection tool, the Chiropractor Interaction and Treatment Equivalence Instrument, may be tailored for specific chiropractic techniques, other manual therapies, and complementary and alternative medicine therapies, and perhaps to interventions delivered by other healthcare providers. The CITE-I also may be useful for several stages of the clinical trial development and implementation process [53,55,62,63]. For example, researchers might use the CITE-I to train clinicians in the delivery of the study protocol in an effort to provide participants in each treatment group with equivalent doses of interactions with the treatment provider, and equivalent treatments when more than one clinician delivers the study treatments [63]. This training procedure might be performed via video-recordings that are either reviewed by the investigators or by the clinicians themselves, to identify areas to treatment standardization (e.g., number of adjustments, clinical information queries) before the start of the trial [62]. Once the trial is underway, the same instrument might be used for quality control purposes to minimize drift in treatment delivery over the course of the trial [64]. Finally, once the trial is concluded, the CITE-I might be used to assess treatment fidelity over the course of the study [53,55].

Our study found potentially important discrepancies in the DC’s verbal interactions, including in communications related to clinical information, explanations, protocol-related directions, and statements about treatment effectiveness between the active and sham groups. In essence, active AMCT participants may have received an ‘augmented interaction’ with the DC, similar to that delivered by acupuncturists in an RCT specifically designed to assess various components of the placebo effect in patients with irritable bowel syndrome [28]. In that study, participants allocated to the augmented interaction group received acupuncturists’ communications that emphasized 5 behaviors shown to support optimal patient-practitioner relationships: a friendly manner, active listening, empathy, thoughtful silence, and communication of confidence in and positive expectations for treatment [28]. These augmented communication styles were not dissimilar to the added interactions the active AMCT participants received when the DC sought more clinical information, offered treatment explanations or self-care recommendations, or shared optimistic statements about participants’ changes in health status. These differences in the practitioner’s verbal interactions may explain the higher satisfaction levels of participants in the active AMCT group reported in the pilot RCT, and possibly account for some of the difference in outcomes between the two chiropractic groups [44].

We also reported the video evaluator’s perceptions of treatment assignment and the RCT participants’ perceptions of treatment believability. The video evaluator correctly attributed ‘active treatment’ to 61% of the active AMCT videos, while incorrectly ascribing ‘placebo/sham’ to only 6% of the active AMCT group. This finding suggests a perceptible difference in the DC’s interactions between treatment groups that allowed a trained evaluator to correctly identify participants who received active AMCT more often than by chance. Similarly, 82% and 93% of active AMCT participants correctly identified their treatment as an active treatment after the first and final treatments, respectively. In contrast, sham AMCT participants shifted their treatment perceptions as inactive from 11% at first treatment to 42% at the final treatment. The video evaluator’s and participants’ perceptions of treatment assignment might be based on two notable differences in treatment delivery identified in this analysis: treatment duration and number of audible sounds generated by the adjusting instrument during its thrust. Active AMCT participants received study visits that were three minutes longer in duration and during which almost twice as many instrument-assisted adjustments were delivered. The sounds made by the adjusting instrument were identified in a previous study as evidence of treatment credibility [37]. Future chiropractic trials with sham treatment groups should develop study protocols that maximize equivalence in such components of treatment delivery.

While our analysis focused on the doctor’s verbal interactions, the non-verbal communications which were not measured in this study may account for the differences noted in the treatment group assignment perceptions of the video evaluator and study participants. Other researchers have identified the importance of such non-verbal communications as tone of voice, facial expression and eye contact [65], the use of touch [65,66], and provider time spent sitting versus standing during clinical encounters [67] on patient satisfaction and health outcomes. Future studies may more closely examine the contributions of non-verbal communication to the placebo effects of chiropractic care although those may be more difficult to adequately record and quantify than were verbal interactions.

Our study has several strengths. Our method of video-recording doctor-patient interactions during chiropractic care is similar to other studies using video-recordings to assess the clinical or communication skills of health professionals [68]. The advantages of video-recordings for this type of research are numerous [69,70]. First, a video-recording is a permanent account of human interactions that are complex, fleeting, and difficult to detail or verify using standard documentation techniques for observational data (e.g., field notes, memos) [69]. As we did during the instrument development process, observers may view video-recorded interactions repeatedly, at different speeds and directions, and with pauses, allowing for thorough and reliable analyses [69,70]. Multiple reviewers also may analyze the same interaction, which may decrease the subjectivity inherent in observational techniques [69,70]. Another strength was the number of recordings analyzed, recorded from multiple participants at different phases of the treatment protocol. In addition, team members were blinded to the treatment assignment of participants throughout the instrument development process as well as during video-recording analysis. These procedures enhance the validity of the study findings [69].

This study had its limitations, including the challenges inherent in the video-recording process [68–71]. Mechanical limitations, such as camera malfunctions, static camera positions, or muffled audio mechanisms, are known issues in research using video-recordings [69–71]. Future studies might position the video-camera on the ceiling, employ two cameras, or use cameras that automatically follow movement to allow fuller visualization of the doctor-patient interaction, as researchers have done in similar studies conducted in emergency departments, physician consultations, or during surgical procedures [64,71,72].

Another limitation is the potential influence of the video-recoding process on the behaviors of the persons whose interactions are recorded [69,70]. Some studies have shown few differences in camera-related behaviors [73] or doctor-patient interactions during video-recorded clinical encounters [68,74], while others indicate improved performance by physicians whose clinical encounters were video-recorded [75]. The frequency with which such behaviors occurred was not evaluated in this analysis, although some patterns were noted that may suggest clinician discomfort with the video-recording process. For example, the camera often was positioned in such a way that it did not visualize the participants’ entire body (most notably the neck and head region) or pick up DC utterances while seated at the head of the treatment table. Future studies should assess clinician comfort with the video-recording process directly.

Another limitation is missing data. We analyzed just 24% of the planned study visits in this pilot RCT. While some missing data-points were from missed appointments, more were from unrecorded treatment visits or incomplete video-recordings. Clinic staff missed or truncated the video-recordings when the office was busy or other clinical demands tasks were prioritized. Similar analyses have reported similar challenges capturing all possible events due to problems with the recording device or human error in initiating the video-recording process [72]. Future studies collecting video-recordings to assess doctor-patient interactions should institute pre-treatment checklists and on-going quality control procedures to assure complete datasets.

In this analysis, we opted not to evaluate the video-recordings for the first treatment visit due to the extended duration and content differences for these visits compared to the T2-T12 study visits. Eight active AMCT participants and 6 sham AMCT participants did not have their T1 study visits video-recorded. Finniss and colleagues note, however, that first treatment encounters may be of critical importance in the “development of subsequent robust placebo responses” (p. 688) through a chain of treatment expectancy, conditioning mechanisms, and the perceived effectiveness of the initial interaction [31]. A future study using this or similar datasets might evaluate the doctor-patient interactions using a more discrete data collection system such as the RIAS to assess group differences in medical history taking, rapport building, self-care instructions, and other socio-emotional relationship components during the initial treatment encounter [49]. Such an evaluation also would allow a comparison of the communication strategies of DCs to other healthcare professionals [76–80].

Lastly, this study was a preliminary investigation of doctor-patient interactions with a pilot clinical trial of chiropractic care. We developed the Chiropractic Interaction and Treatment Equivalence Instrument specifically for this preliminary study. While the conceptual framework for the instrument seems logical and our analysis did identify differences in the doctor’s interactions between treatment groups, the CITE-I requires further refinement, including formal instrument testing to establish its reliability and validity. Item analysis may identify different domains than those presented here, as well as individual items that are redundant or that might be omitted. Psychometric evaluations of the CITE-I should occur before its use in other clinical studies of chiropractic care or in other manual therapy trials.

## Conclusion

Our findings show the feasibility of evaluating doctor-patient verbal interactions and treatment equivalence in chiropractic clinical trials using video-recordings of doctor-patient encounters and a standardized data collection tool, the Chiropractor Interaction and Treatment Equivalence Instrument. The results of our study indicated that doctor-patient interactions in randomized controlled trials of chiropractic therapies may vary between the active care and sham-controlled treatment groups. It is not known how much effect such variation in doctor-patient interaction has on clinical outcomes. However, to accurately compare the clinical value of one form of treatment to that of another, clinical trial design and training protocols of clinicians who deliver study interventions should include steps to minimize the variation of doctor-patient interactions between treatment groups. Future studies to establish the psychometric properties of the CITE-I are needed.

## Abbreviations

AAI: Activator adjusting instrument
AMCT: Activator methods chiropractic technique
CAM: Complementary and alternative medicine
CITE-I: Chiropractor interaction and treatment equivalence instrument
DC: Doctor of chiropractic
ID: Identification number
RCT: Randomized controlled trial
RIAS: Roter interaction analysis system
T: Treatment (number)
TMD: Temporomandibular joint disorder
